# Oxidized Phosphatidylcholines Regulate Secretory Phospholipase A_2_ Through Membrane Nanodomain Remodeling

**DOI:** 10.3390/molecules31081298

**Published:** 2026-04-16

**Authors:** Vesela Yordanova, Rusina Hazarosova, Victoria Vitkova, Ralitsa Angelova, Biliana Nikolova, Atanaska Elenkova, Albena Momchilova, Galya Staneva

**Affiliations:** 1 Institute of Biophysics and Biomedical Engineering, Bulgarian Academy of Sciences, Acad. Georgi Bonchev Str., bl. 21, 1113 Sofia, Bulgaria; vyordanova@biophys.bas.bg (V.Y.); rhazarosova@biophys.bas.bg (R.H.); ralitsa.angelova@biophys.bas.bg (R.A.); nikolova@bio21.bas.bg (B.N.); albena_momchilova@abv.bg (A.M.); 2 Department of Endocrinology, Faculty of Medicine, Medical University-Sofia, 15 Acad. Ivan Geshov Blvd., 1000 Sofia, Bulgaria; atanaskae@gmail.com; 3 Institute of Solid State Physics, Bulgarian Academy of Sciences, 72 Tzarigradsko Chaussee Blvd., 1784 Sofia, Bulgaria; victoria@issp.bas.bg

**Keywords:** oxidized phospholipids, membrane lateral organization, lipid rafts, sPLA_2_ activity, docosahexaenoic acid, cholesterol

## Abstract

Oxidative stress generates oxidized phospholipids (OxPLs) that alter membrane structure and inflammatory lipid signaling, yet the underlying biophysical mechanisms remain poorly understood. Here, we examine how two structurally distinct truncated oxidized phosphatidylcholines (OxPCs), 1-palmitoyl-2-(5′-oxo-valeroyl)-*sn*-glycero-3-phosphocholine (POVPC) and 1-palmitoyl-2-glutaryl-*sn*-glycero-3-phosphocholine (PGPC), remodel membrane lateral organization and regulate secretory phospholipase A_2_ (sPLA_2_) activity. Large unilamellar vesicles composed of sphingomyelin, cholesterol, and either monounsaturated 1-palmitoyl-2-oleoyl-*sn*-glycero-3-phosphocholine (POPC) or polyunsaturated 1-palmitoyl-2-docosahexaenoyl-*sn*-glycero-3-phosphocholine (PDPC) were used to reconstitute the liquid-ordered/liquid-disordered (L_o_/L_d_) phase coexistence characteristic of eukaryotic plasma membranes. Fluorescence spectroscopy revealed that OxPLs modulate lipid packing and nanodomain organization in a structure- and composition-dependent manner. POVPC promoted pronounced membrane ordering and L_o_ domain stabilization compared with PGPC, particularly in monounsaturated membranes with low cholesterol content. In contrast, PDPC-containing membranes, especially at elevated cholesterol, exhibited enhanced structural resilience to OxPL-induced perturbations. These biophysical changes were associated with distinct functional outcomes. Notably, the relationship between membrane structural parameters and sPLA_2_ activity was not linear, indicating a decoupling between bulk membrane properties and enzymatic response. sPLA_2_ activity was linked to membrane lateral organization: the size of L_o_ domains modulate hydrolysis by influencing the physicochemical properties of L_o_/L_d_ interfaces, which may represent preferential sites for enzyme activation. Consistent with this, POVPC reduced sPLA_2_ activity through stabilization of ordered domains at both low and high cholesterol, while PGPC enhanced hydrolysis at high cholesterol. Importantly, PDPC-containing membranes attenuated sPLA_2_ activity and exhibited a protective effect against OxPC-induced enzymatic activation. Together, these findings identify membrane lateral organization as a key regulator of sPLA_2_ function and provide mechanistic insight into how oxidative stress can differentially modulate inflammatory lipid signaling depending on membrane composition. This work highlights membrane organization as an active determinant of enzyme activity and a potential target in pathologies associated with oxidative stress, including atherosclerosis, neuroinflammation, and metabolic disease.

## 1. Introduction

Biological membranes are dynamic structures that maintain cellular integrity and organize essential processes such as signaling, transport, and metabolism [1]. Their function is governed by the physicochemical properties of the lipid bilayer, primarily composed of glycerophospholipids (GPLs), sphingolipids (SLs), and cholesterol (Chol) [2]. Chol plays a central role in modulating membrane organization, lipid packing, and dynamic behavior, and drives the formation of lateral heterogeneities through preferential interactions with saturated lipids [3]. This results in the coexistence of liquid-ordered (L_o_) domains known as lipid rafts enriched in Chol and sphingomyelin (SM) and more fluid non-raft liquid-disordered (L_d_) regions rich in unsaturated phospholipids (PLs) [4]. Such domain organization is critical for various membrane-related processes, including signal transduction, cell adhesion, membrane trafficking, and metabolic regulation [4,5].

**Figure 1:**
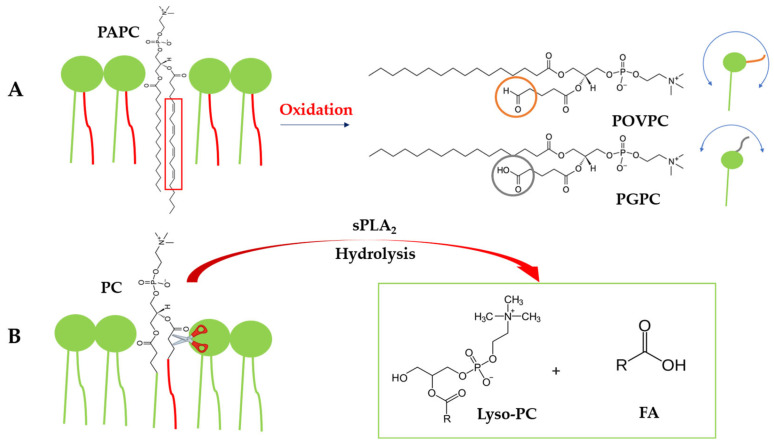
Schematic representation of oxidized phospholipid formation and sPLA_2_-mediated hydrolysis. (**A**) Oxidation of the *sn-2* acyl chain of PAPC (highlighted in red box) yields POVPC (aldehyde-terminated, in orange circle) and PGPC (carboxyl-terminated, in grey circle). POVPC adopts multiple *sn*-2 chain orientations (in orange color with blue bidirectional arrow illustrating its movement), including configurations toward the aqueous phase and along the membrane interface, whereas PGPC shows a more pronounced outward orientation of its oxidized chain (in grey color with blue bidirectional arrow illustrating its movement) [8]. (**B**) Secreted phospholipase A_2_ (sPLA_2_) hydrolyzes the *sn-2* ester bond (depicted with scissors and red arrow), producing lysophosphatidylcholine (Lyso-PC) and a free fatty acid (FA) (in green box).

Phosphatidylcholines (PCs) are the dominant membrane lipids, typically containing a saturated fatty acid (FA) at the *sn-1* position and an unsaturated fatty acid at the *sn-2* position [6]. Polyunsaturated fatty acids (PUFAs), particularly the Ω-3 docosahexaenoic acid (DHA), enhance membrane fluidity and are essential for proper cellular function, but their high degree of unsaturation makes them especially susceptible to oxidative damage [7]. Lipid peroxidation leads to the formation of oxidized phospholipids (OxPLs), which are established mediators of inflammation and cellular dysfunction, and are implicated in pathologies such as atherosclerosis, cancer, and neurodegeneration. Among these, truncated oxidized phosphatidylcholines (OxPCs), including 1-palmitoyl-2-(5′-oxo-valeroyl)-*sn*-glycero-3-phosphocholine (POVPC) and 1-palmitoyl-2-glutaryl-*sn*-glycero-3 phosphocholine (PGPC) derived from 1-palmitoyl-2-arachidonoyl-phosphatidylcholine (PAPC) (Figure 1). Their conformation within membranes depends on the terminal functional groups, with POVPC adopting multiple *sn-2* chain orientations, including configurations toward the aqueous phase and along the membrane interface. Notably, PGPC shows a more pronounced outward orientation of its oxidized chain [8]. These features enable altered lipid packing and promote interactions with cellular receptors and inflammatory pathways [9].

Oxidative modification of PLs is also known to disrupt membrane organization and to influence the activity of interfacially active enzymes such as secretory phospholipase A_2_ (sPLA_2_), a key regulator of lipid metabolism and inflammation [10]. sPLA_2_ catalyzes the hydrolysis of the *sn-2* ester bond, producing lysophosphatidylcholine (lyso-PC) and free fatty acids (Figure 1). Together, these processes link oxidative lipid modification to changes in membrane organization and enzymatic activity. Importantly, sPLA_2_ activity depends strongly on membrane composition, lateral structure, and physical state, all of which are modulated by Chol content and distribution [11].

Despite these advances, a mechanistic understanding of how specific OxPCs, such as POVPC and PGPC, perturb Chol-dependent membrane organization is still lacking, particularly in systems exhibiting L_o_/L_d_ phase coexistence. In addition, it remains unclear how these structural changes influence sPLA_2_ activity, and how this interplay is modulated by lipid unsaturation, especially in membranes containing highly unsaturated DHA-rich PLs.

Here, we address these questions using liposome model membranes composed of ternary PC:SM:Chol mixtures that reproduce L_o_/L_d_ coexistence. By comparing unsaturated lipid species only differing in FA at the *sn-2* position of the PC molecule—monounsaturated oleic acid (OA, C18:1 ∆^9^, the most common unsaturated FA) in 1-palmitoyl-2-oleoyl-*sn*-glycero-3-phosphocholine (POPC) and polyunsaturated docosahexaenoic acid (DHA, C22:6 ∆^4,7,10,13,16,19^, the longest and most unsaturated FA belonging to the family of Ω-3 fatty acids) in 1-palmitoyl-2-docosahexaenoyl-*sn*-glycero-3-phosphocholine (PDPC) in the presence of POVPC and PGPC—we directly examine how fatty acid unsaturation, OxPCs, and Chol collectively regulate membrane organization and sPLA_2_ activity, providing new insight into the molecular basis of oxidized lipid-induced membrane remodeling.

## 2. Results

**Figure 2:**
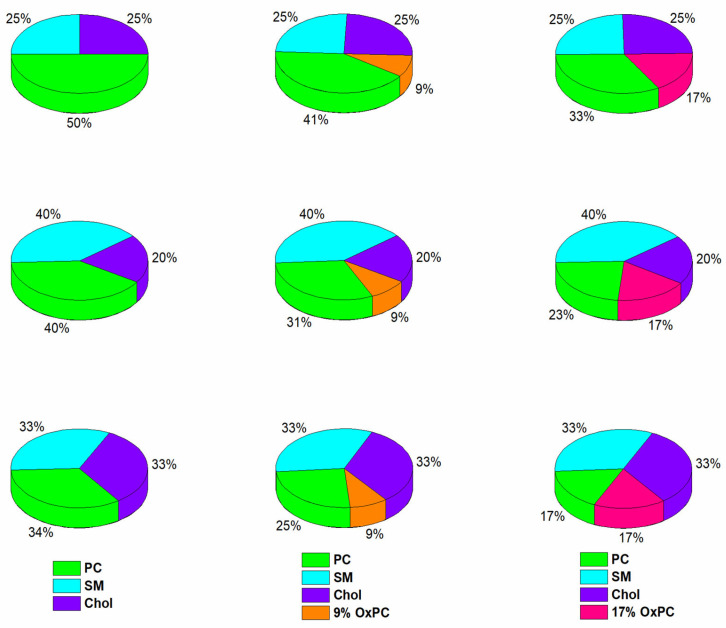
Pie charts representing the lipid compositions of mono- or polyunsaturated membrane models used in this study: left column—control ternary PC:SM:Chol systems with molar ratios 50:25:25 (1:0.5:0.5), 40:40:20 (1:1:0.5) and 34:33:33 (1:1:1); middle column—quaternary systems with 9 mol% OxPC (POVPC or PGPC); right column—quaternary systems with 17 mol% OxPC.

Of particular interest in our study was the combined effect of oxidized lipids (POVPC or PGPC) and DHA at the *sn-2* position of the PC molecule (PDPC) on lateral membrane organization and sPLA_2_ activity compared to monounsaturated PC (POPC). In addition to that, Chol, as a central component of cell membranes, plays a critical role in maintaining membrane integrity and organization. Typically present at 20–50% in mammalian membranes [12], it is essential for the formation and stability of lipid rafts. Consequently, studying the interactions of Chol-containing membranes with oxidized lipids is of great importance. The effect of OxPCs was studied in ternary POPC:SM:Chol and PDPC:SM:Chol mixtures modeling the formation of domains in L_o_ phase, surrounded by a lipid matrix in L_d_ phase, and representing L_o_/L_d_ coexistence to mimic the cell plasma membranes. To study the membrane organization, we prepared PC:SM:Chol large unillamelar vesicles (LUVs) in 50:25:25, 40:40:20 and 34:33:33 molar ratios in the absence and presence of OxPCs (9 and 17 mol%). The concentration of OxPC (POVPC or PGPC) replaced certain amounts of PC (POPC or PDPC) in control vesicles in order to mimic the generation of oxidized lipids in the membrane (Figure 2). The sPLA_2_ activity was measured in the following mixtures: ternary PC:SM:Chol at molar lipid ratios of 100:50:50, 100:100:50 and 100:100:100 (controls), and quaternary PC:SM:Chol:OxPC 100:50:50:30, 100:100:50:30 and 100:100:100:30. The aim was to keep the concentration of PC molecules constant, as they are PLA_2_ substrates and thus the substrate:enzyme ratio would always be the same. However, in all studied ternary LUVs, the lipid ratios were 1:0.5:0.5, 1:1:0.5 and 1:1:1 (mol/mol), modeling a SM:Chol ratio of 1:1 or 2:1, which is consistent with that found in cellular raft domains. It did not change in the studied control ternary systems, in comparison to the respective OxPC-containing quaternary ones.

### 2.1. Effect of OxPCs on the Temperature Dependence of Laurdan Generalized Polarization (GP) in the Ternary PC:SM:Chol Mixtures (Model of L_o_/L_d_ Coexistence)

**Figure 3:**
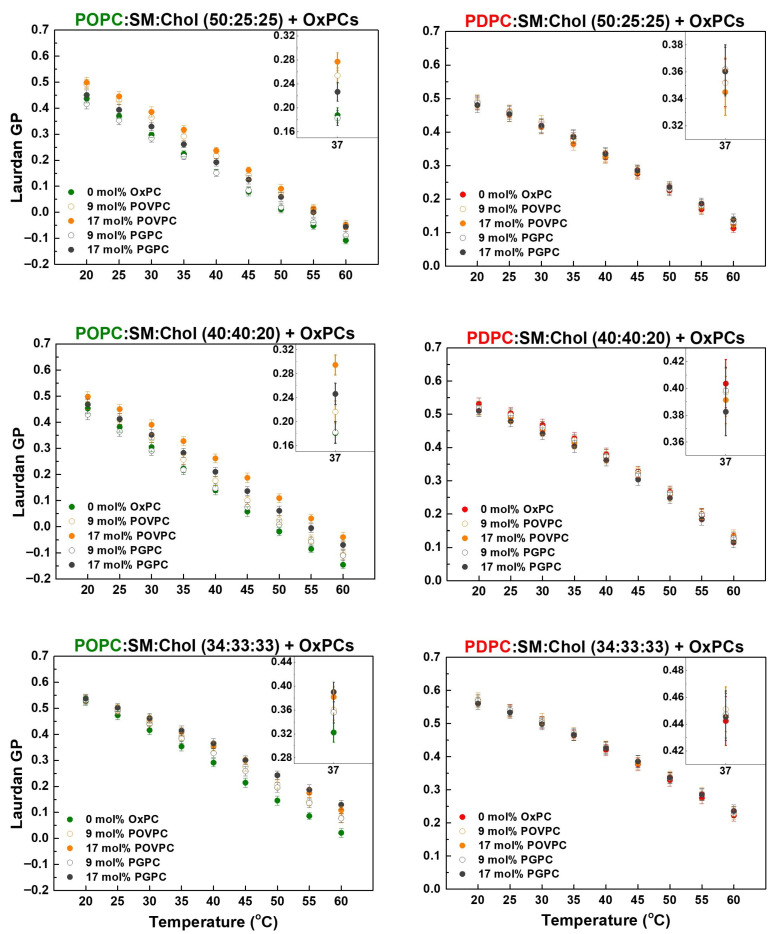
Laurdan GP as a function of temperature (from 20 to 60 °C) in monounsaturated POPC:SM:Chol (green font) and polyunsaturated PDPC:SM:Chol (red font) LUVs (50:25:25, 40:40:20, 34:33:33) in the absence and presence of OxPC (9 and 17 mol%). Inset: GP values calculated for physiological temperature, 37 °C. The data represent means ± SD from 3 experiments with 3 measurements per point.

The effect of OxPCs (POVPC or PGPC) on the membrane lipid order depending on the degree of fatty acid unsaturation at the *sn-2* position of PC molecules in bilayers was studied at temperatures ranging from 20 to 60 °C by using Laurdan fluorescence spectroscopy. The absolute GP values, calculated from the recorded emission spectra, represent an indirect quantitative measure of the membrane lipid order. Figure 3 shows a gradual GP decrease with increasing temperature in all studied lipid mixtures. It is well known that Chol increases lipid-packing density and maintains membrane fluidity. Therefore, the membranes with higher Chol content (33 mol%; GP_POPC:SM:Chol_ = 0.32, GP_PDPC:SM:Chol_ = 0.44) were more ordered than ones with lower Chol content (25 mol%; GP_POPC:SM:Chol_ = 0.19, GP_PDPC:SM:Chol_ = 0.35) in systems with an equimolar SM:Chol ratio at 37 °C (Figure 3). In the third studied mixture (40:40:20), characterized by a SM:Chol ratio of 2:1 and the lowest Chol content (20 mol%; GP_POPC:SM:Chol_ = 0.18, GP_PDPC:SM:Chol_ = 0.40), OxPCs exerted the strongest effect on membrane lateral organization (Figure 3, Inset).

OxPCs in the monounsaturated POPC:SM:Chol matrix increased the lipid order, i.e., exhibited a concentration-dependent rigidifying effect. It is also clearly seen that in vesicles with the highest Chol content (34:33:33), the OxPC effect increased with increasing temperature. POVPC increased lipid order to a greater extent than PGPC in POPC:SM:Chol LUVs with lower Chol concentrations (20 and 25 mol%), while in vesicles with high Chol content (33 mol%), the effect of both OxPCs was equal. However, in the polyunsaturated PDPC:SM:Chol membranes only a minor change in lipid ordering was observed in OxPC-containing LUVs, as a barely noticeable fluidizing effect could be seen in the mixture with the lowest Chol content (40:40:20) (Figure 3).

**Figure 4:**
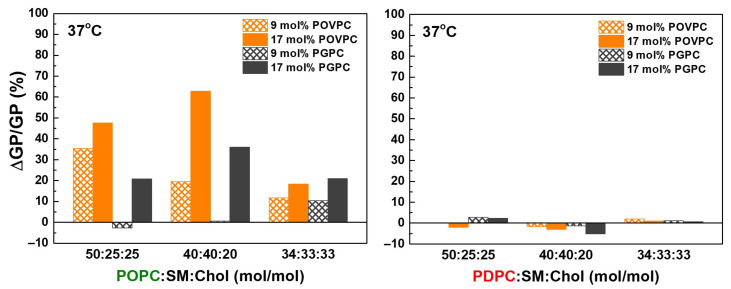
The relative GP change (%), ΔGP/GP = ((GP_OxPC_ − GP)/GP) ∗ 100, where GP_OxPC_ denotes the value in the OxPC-containing sample, and GP stands for the same sample without OxPC (control) at 37 °C. ΔGP/GP (%) quantified the effect of the oxidized lipids (POVPC or PGPC) on lipid order in ternary PC:SM:Chol mixtures depending on the degree of unsaturation at the *sn-2* position of PC molecules (POPC, in green font or PDPC, in red font).

Figure 4 shows that the two investigated OxPCs had the strongest effect on both lipid matrices at a SM:Chol ratio of 2:1, i.e., at the lowest Chol concentration (20 mol%). Furthermore, lipid order at the glycerol level was significantly more affected by OxPCs in monounsaturated membranes than in polyunsaturated ones.

### 2.2. Effect of OxPCs on Formation and Size of Raft-like Domains in the Ternary PC:SM:Chol Mixtures (Model of L_o_/L_d_ Coexistence)

**Figure 5:**
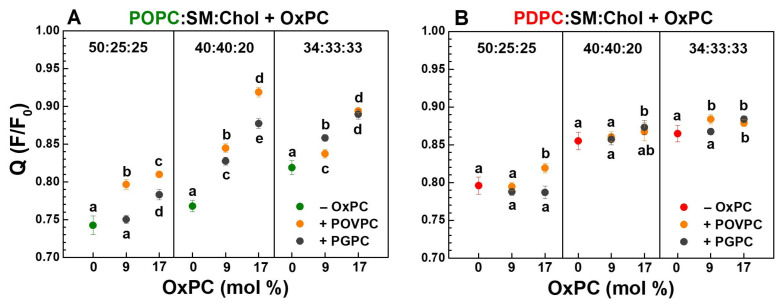
Effect of OxPCs on DPH fluorescence quenching in the presence of TEMPO (2 mM), Q = F/F_0_, as a function of OxPC (POVPC or PGPC) concentration in monounsaturated POPC:SM:Chol (green font) (**A**) and polyunsaturated PDPC:SM:Chol (red font) (**B**) LUVs at 37 °C. The data represent means ± SD from 3 experiments with 3 measurements per point (n = 9). One-Way ANOVA analysis was performed to compare control ternary PC:SM:Chol samples in each molar ratio with the corresponding OxPC-containing quaternary ones, as well as POVPC-containing samples with PGPC-containing ones, in each concentration (9 and 17 mol%). Vertical selections were used to separate the comparison groups for clarity. The population data followed a normal Gaussian distribution. Groups sharing at least one common letter are not significantly different from each other, whereas groups labeled with different letters are significantly different.

To clarify the influence of OxPCs (9 and 17 mol%) on the formation of raft-like (L_o_) domains, we used the DPH-TEMPO quenching method (Figure 5). OxPCs reduced DPH fluorescence quenching in a concentration-dependent manner, with a stronger effect observed in the monounsaturated matrix compared to the polyunsaturated one. In monounsaturated quaternary OxPC-containing mixtures, Q values increased in comparison to control ternary POPC:SM:Chol ones which corresponds to an increase in L_o_ domain formation (Figure 5A). In vesicles with lower Chol concentrations (20 and 25 mol%), POVPC enhances the ordered phase formation to a greater extent than PGPC, while in high Chol content (33 mol%) there was no statistically significant difference in the effect of both studied OxPCs. This result is consistent with the OxPC influence on lipid order at the glycerol level in Laurdan-labeled LUVs (Figure 3 and Figure 4). In the polyunsaturated matrix, OxPCs have a different but significantly weaker effect (up to 3%) on L_o_ domain formation (Figure 5B).

Q values of the “pure” lipid phases, which are needed to calculate L_o_ domain radii were as follows: Q_POPC_ = 0.60 for single-component POPC vesicles and Q_PDPC_ = 0.58 for PDPC ones as a model of the L_d_ phase, and Q_SM:Chol_ = 0.93 for binary SM:Chol mixture as a model of the L_o_ phase. Pure PC vesicles show different values of the Q parameter. This arises from the different structural characteristics of the two types of PC molecules (POPC and PDPC) included in the studied mixtures [13].

**Figure 6:**
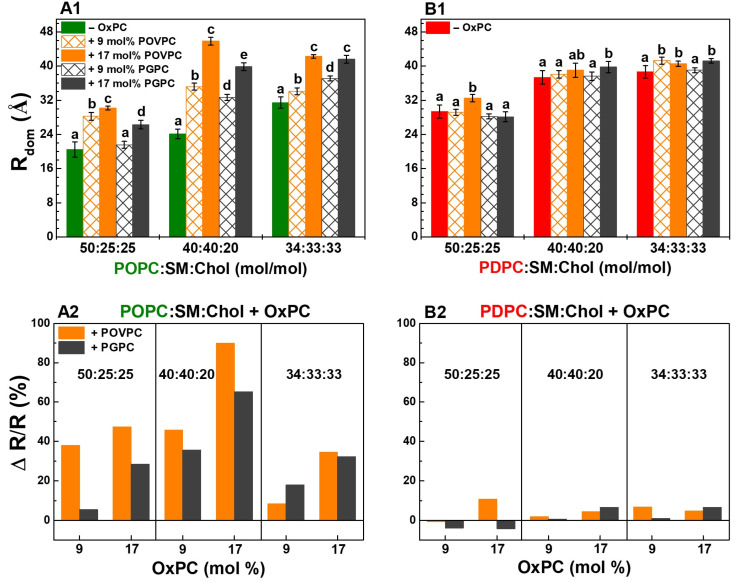
(1) Nanodomain radius (R_dom_, Å) in monounsaturated POPC:SM:Chol (green font) (**A1**) and polyunsaturated PDPC:SM:Chol (red font) (**B1**) matrix as a function of lipid mixtures at 37 °C. The calculated data, based on the DPH-TEMPO quenching method, represent means ± SD (n = 9). One-Way ANOVA analysis was performed to compare control ternary PC:SM:Chol samples in each molar ratio with the corresponding OxPC-containing quaternary ones, as well as POVPC-containing samples with PGPC-containing ones, in each concentration (9 and 17 mol%). Minor ticks on the X-axis were used to separate the comparison groups for clarity. The population data followed a normal Gaussian distribution. Groups sharing at least one common letter are not significantly different from each other, whereas groups labeled with different letters are significantly different. (2) The relative R_dom_ change (%), ΔR/R = ((R_OxPC_ − R)/R) ∗ 100, where R_OxPC_ denotes the value in OxPC-containing sample, and R stands for the same sample without OxPC (control). ΔR/R (%) quantifies the effect of the oxidized lipids (POVPC or PGPC) on domain size in ternary PC:SM:Chol mixtures (**A2**,**B2**).

The polyunsaturated matrix promoted the formation of larger nanodomains, with sizes ranging from 29 to 39 Å, whereas the monounsaturated matrix resulted in smaller nanodomains between 20 and 31 Å. This indicates that the degree of unsaturation in the lipid matrix influences nanodomain size, with higher unsaturation favoring the formation of larger domains. In the polyunsaturated matrix (Figure 6(B1)), the smallest L_o_ domains in 50:25:25 PDPC:SM:Chol are close in size to the largest domains in the monounsaturated matrix (Figure 6(A1)) in 34:33:33 POPC:SM:Chol and about 8 Å smaller than the domains of the other two PDPC:SM:Chol mixtures studied. The largest difference in the nanodomain size between the two types of matrices was found at a lipid ratio of 40:40:20 (13 Å).

OxPCs stimulate the formation of raft domains with larger sizes. POVPC affected domain size most strongly in the 40:40:20 POPC:SM:Chol mixture and least in 34:33:33 POPC:SM:Chol, i.e., the lower the Chol concentration, the greater the POVPC effect was (Figure 6(A2)). An interesting result was that the largest domain size (46 Å) was calculated in a monounsaturated matrix 23:17:40:20 POPC:POVPC:SM:Chol (Figure 6(A1)). The presence of 17 mol% POVPC increased the radius of the L_o_ nanodomains (R_dom_) by 90% in the 40:40:20 POPC:SM:Chol mixture, 25% more than PGPC (Figure 6(A2)). In the polyunsaturated matrix, the largest increase (10%) was seen for 33:17:25:25 PDPC:POVPC:SM:Chol (Figure 6(B2)). The 17:17:33:33 PC:OxPC:SM:Chol mixtures showed the same domain radius (41 Å) (Figure 6(A1,B1)), with no significant difference between POVPC and PGPC, though the relative increase compared to controls was six times higher in POPC-containing LUVs (≈30%) than in PDPC-containing ones (5%) (Figure 6(A2,B2)). It should still be noted that the presented domain radius values are indicative, as the domains may have irregular shapes [14].

### 2.3. Effect of OxPCs on sPLA_2_ Activity in the Ternary PC:SM:Chol Mixtures (Model of L_o_/L_d_ Coexistence)

**Figure 7:**
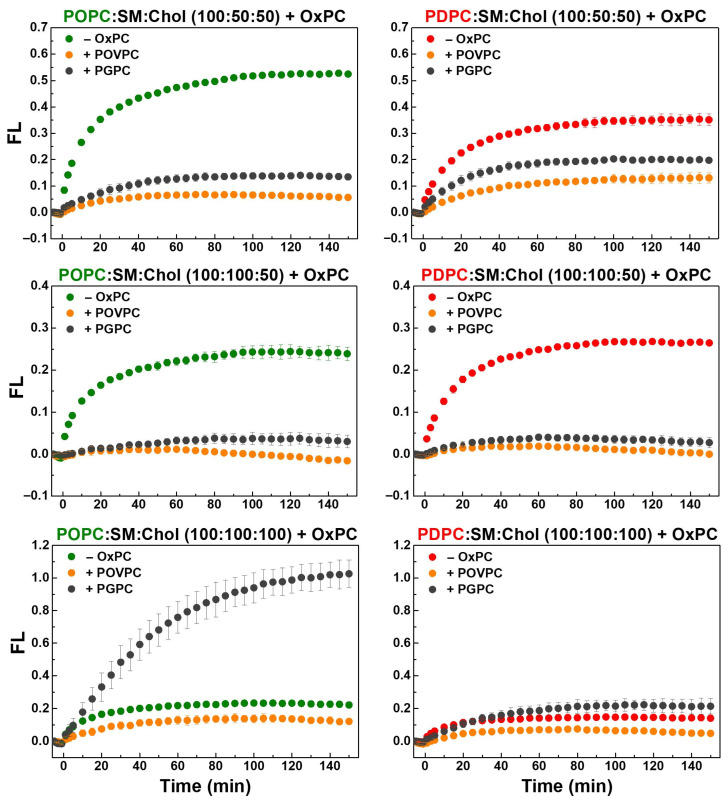
Kinetic curves of hydrolysis of PED6 incorporated in control monounsaturated (green font) and polyunsaturated (red font) ternary PC:SM:Chol LUVs (100:50:50, 100:100:50, 100:100:100) and OxPC-containing quaternary ones (OxPC = 30 mol% POVPC or PGPC) by sPLA_2_ at 37 °C. PC/PED6 and (PC + PED6)/enzyme ratios were 10:1 mol/mol and 8000:1 mol/mol. FL = (F_530_/F_530, initial_) − 1, where F_530_ was the fluorescence (FL) intensity at 530 nm at time t, whereas F_530, initial_ represented the sample FL intensity before bvPLA_2_ addition (at t = 0 min). FL signal increase was correspondent to sPLA_2_ activity elevation. FL values were presented at every 5 min for clarity. The data represent means ± SD from 3 experiments, as each sample was measured 4 times (n = 12). One-Way ANOVA analysis was performed (Figure S3).

The effect of truncated OxPCs (30 mol%) on sPLA_2_ activity in ternary POPC:SM:Chol and PDPC:SM:Chol vesicles at lipid ratios of 100:50:50, 100:100:50 and 100:100:100, as determined using a fluorogenic PLA_2_ assay, is presented in Figure 7. sPLA_2_ activity was the highest in membranes with low SM and Chol content (Figure S1), and was mostly unaffected by Chol in monounsaturated membranes (Figure S1A), while polyunsaturated membranes showed reduced enzymatic activity at high SM and Chol levels (Figure S1B).

As shown in Figure 7, the aldehydoacyl lipid POVPC consistently inhibited sPLA_2_ activity in all ternary mixtures, whereas the carboxyacyl lipid PGPC had a variable effect—weakly inhibitory at lower SM and Chol levels but enhancing enzymatic activity in the 100:100:100 mixtures.

**Figure 8:**
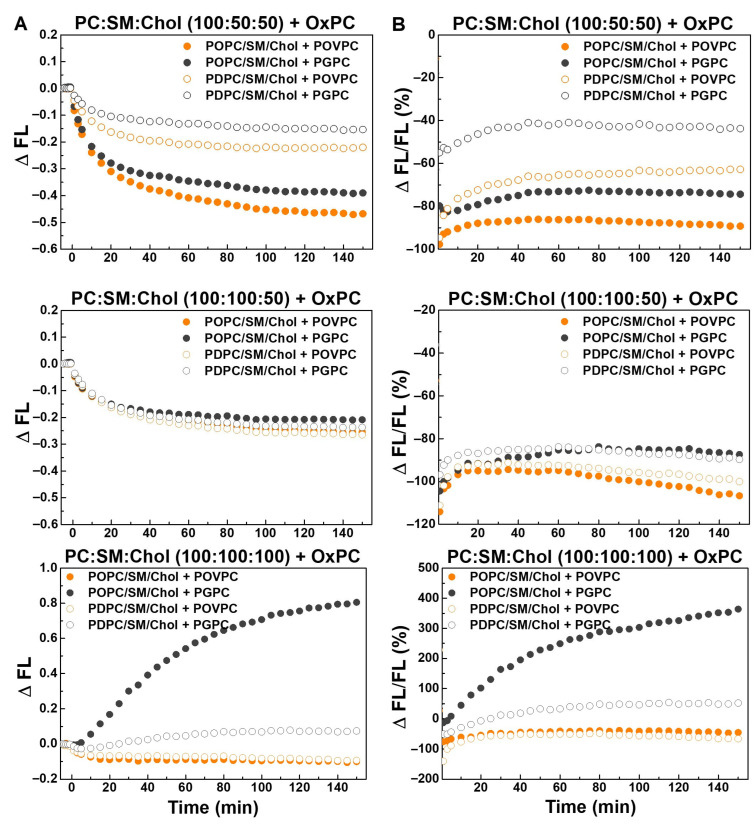
Effect of truncated OxPCs (30 mol% POVPC or PGPC) on sPLA_2_ activity in PED-labeled monounsaturated POPC:SM:Chol and polyunsaturated PDPC:SM:Chol LUVs shown as both absolute (**A**) and relative (**B**) changes as a function of time at 37 °C. (**A**) The absolute change, ∆FL = FL_OxPC_ − FL, where FL_OxPC_ denotes the value in OxPC-containing sample, and FL stands for the same sample without OxPC (control). (**B**) The relative FL change (%), ΔFL/FL = ((FL_OxPC_ − FL)/FL) ∗ 100, for OxPC-containing quaternary mixtures.

Figure 8 shows that at a SM:Chol ratio of 2:1, OxPCs strongly inhibited sPLA_2_ activity, with POVPC causing complete inhibition and PGPC nearly 90%. In 100:50:50 mixtures, inhibition was 1.5 times stronger in monounsaturated membranes, with POVPC more effective than PGPC. In equimolar mixtures, POVPC had a stronger inhibitory effect in polyunsaturated membranes, whereas PGPC markedly stimulated sPLA_2_ activity in monounsaturated membranes.

**Figure 9:**
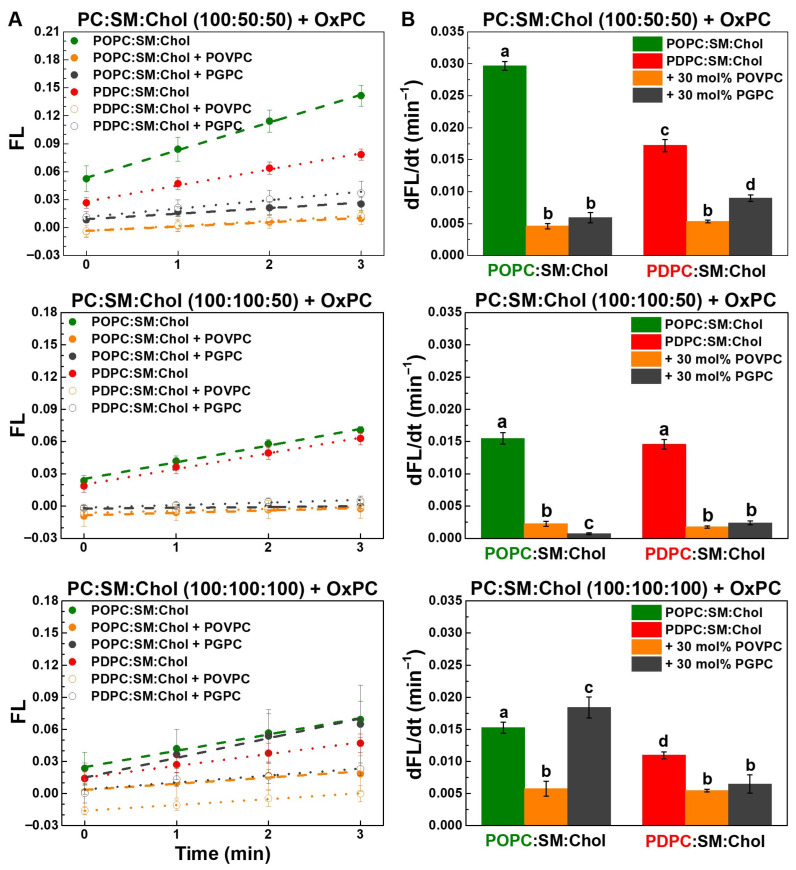
The reaction rate of hydrolysis of PED6 incorporated in control monounsaturated (green font) and polyunsaturated (red font) ternary PC:SM:Chol LUVs (100:50:50, 100:100:50, 100:100:100) and OxPC-containing quaternary ones (OxPC = 30 mol% POVPC or PGPC) by sPLA_2_ at 37 °C. Linear regression of the initial part of the kinetic curves of normalized FL intensity, FL, at 530 nm (FL = F_530_/F_530, initial_ − 1) = *a* + *b*t, where *a* and *b* denote the y-intercept (**A**) and the slope (**B**), respectively. The data represent means ± SD from 3 experiments, as each sample was measured 4 times (n = 12). (**A**) The intercept yielded the FL value at t = 0 min. (**B**) The enzymatic reaction rate, dFL/dt (min^−1^), was determined as the slope of the kinetic curves. One-Way ANOVA analysis was performed. The population data followed a normal Gaussian distribution. Groups sharing at least one common letter are not significantly different from each other, whereas groups labeled with different letters are significantly different.

The highest sPLA_2_ reaction rate was observed in control monounsaturated POPC:SM:Chol 100:50:50 membranes (Figure 9 and Figure S2), while it was roughly halved in 100:100:50 and 100:100:100 POPC-containing mixtures, reduced threefold in PDPC:SM:Chol 100:100:100, and lowest in quaternary PC:SM:Chol:OxPC 100:100:50:30 mixtures (Figure 9). OxPCs had a pronounced impact on the enzymatic reaction rate, leading to an overall substantial reduction. In POPC:SM:Chol:OxPC 100:50:50:30 mixtures, the reaction rate was reduced by approximately 80–85% compared to the control. In polyunsaturated membranes with the same lipid ratio, the decrease was 70% in POVPC-containing vesicles and 50% in PGPC-containing ones. In vesicles with an equimolar lipid ratio, OxPCs exhibited a weaker inhibitory effect in polyunsaturated membranes, reducing the reaction rate by 50% and 40%, respectively. In monounsaturated membranes, POVPC decreased the reaction rate by about 60%, whereas PGPC led to a 20% increase compared to the control.

It should be noted that the kinetic profiles may be influenced by the accumulation of hydrolysis products during the reaction. The generation of lysophospholipids and free fatty acids is expected to alter membrane properties, including lipid packing and lateral organization, which may affect enzyme activity over time. Thus, the observed kinetics likely reflect both the initial membrane composition and changes occurring during the reaction.

In summary of the presented results (Table 1), PGPC exhibits a Chol-dependent dual behavior, switching from an inhibitor at low Chol to a strong activator of sPLA_2_ in Chol-rich membranes. The most pronounced effect is observed in monounsaturated (POPC) systems at high Chol, where PGPC induces a ~350% increase in enzymatic activity. In contrast, DHA-containing (PDPC) membranes markedly attenuate this activation, reducing the effect of PGPC by approximately 300%. Notably, POVPC remains inhibitory under all tested conditions and exhibits a stronger inhibitory effect on sPLA_2_ activity than PGPC. Furthermore, the inhibitory effect of POVPC is more pronounced in monounsaturated (POPC) membranes than in polyunsaturated (PDPC) systems, indicating that lipid unsaturation mitigates the impact of OxPC on enzyme activity.

Importantly, these functional changes in sPLA_2_ activity are accompanied by alterations in membrane physical properties; however, they do not correlate in a direct or linear manner, revealing a clear decoupling between enzymatic response and bulk membrane parameters. Both OxPCs increase membrane order in POPC-containing systems, with a markedly stronger effect observed for POVPC (up to ~+48% at low Chol), whereas no significant changes are detected in PDPC membranes. Similarly, the size of liquid-ordered (L_o_) domains is increased in POPC systems, particularly in the presence of POVPC, while PDPC-containing membranes exhibit only minor or even slightly negative changes (e.g., PGPC at low Chol). At high Chol, PGPC-induced activation of sPLA_2_ occurs despite only moderate increases in membrane order (~+20%) and L_o_ domain size (~+32%), further supporting the absence of a simple linear relationship and suggesting that local membrane heterogeneities or packing defects may play a dominant role. In contrast, the consistently inhibitory behavior of POVPC coincides with stronger effects on membrane ordering and domain size.

Overall, these findings demonstrate that the effects of oxidized phospholipids on sPLA_2_ activity are strongly modulated by Chol content and lipid unsaturation, reflecting a complex and non-linear interplay between membrane composition, physical properties, and enzyme–membrane interactions.

## 3. Discussion

**Figure 10:**
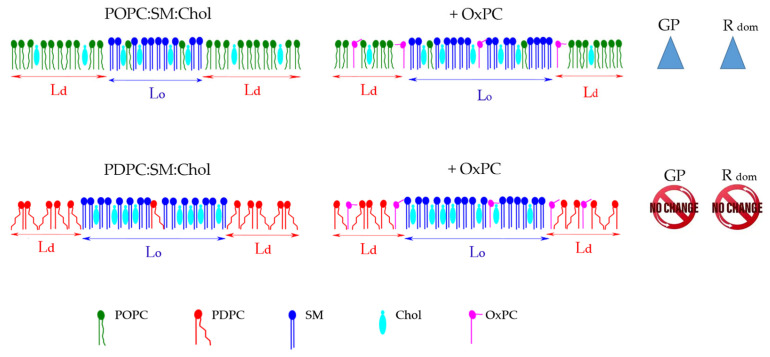
Schematic representation of OxPC-induced modulation of membrane lateral organization in mono- and polyunsaturated lipid matrices. Membranes composed of POPC:SM:Chol (top) and PDPC:SM:Chol (bottom) exhibit liquid-ordered (L_o_) and liquid-disordered (L_d_) phase coexistence. PDPC-containing membranes display larger L_o_ domains compared to POPC ones. The incorporation of oxidized phospholipids (OxPCs) in POPC-containing membranes increases lipid order (GP) and domain size (R_dom_). In contrast, PDPC-containing membranes show minimal changes upon OxPC addition. Color coding: POPC (green), PDPC (red), SM (blue), Chol (cyan), and OxPCs (magenta).

The present study demonstrates that the effects of truncated OxPCs on membrane organization are strongly dependent on the lipid environment, particularly the degree of fatty acid unsaturation and cholesterol content. This concept is summarized schematically in Figure 10, which highlights the distinct responses of mono- and polyunsaturated membranes to oxidative perturbations. A key finding is that OxPCs exert a stronger effect on membrane order and nanodomain size in monounsaturated POPC-based ternary mixtures than in polyunsaturated PDPC-based ones, indicating that polyunsaturated matrices intrinsically buffer oxidative perturbations. Consistently, PDPC promotes the formation of larger raft domains compared to POPC, as illustrated in Figure 10. This behavior is attributed to the reduced miscibility of SM and Chol within the polyunsaturated matrix. As raft domains are primarily enriched in SM and Chol, the presence of highly polyunsaturated acyl chains disfavors their mixing with the surrounding lipids relative to monounsaturated systems, thereby enhancing lateral segregation and leading to the formation of larger L_o_ domains.

Our Laurdan and DPH–TEMPO data are consistent with previously reported data [15] and support a model in which PDPC modulates the partitioning of SM and Chol between L_o_ and L_d_ phases, promoting tighter L_o_ lipid packing and stabilization of larger L_o_ domains [15]. This interpretation is further supported by solid-state ^2^H NMR studies demonstrating increased domain size in PDPC-containing membranes, consistent with models in which DHA regulates raft architecture [16].

Chol also plays a protective role against OxPC-induced structural perturbations, particularly in monounsaturated membranes, where increasing Chol content attenuates the effects of oxidized lipids on membrane order and nanodomain organization. Under these conditions, POVPC exerts a stronger structural impact than PGPC, especially at lower Chol levels. This behavior is consistent with biophysical studies showing that Chol preferentially interacts with the carbonyl groups of oxidized lipid chains, facilitating the reorganization of damaged regions rather than simply increasing membrane condensation [17]. From a structural perspective, Chol’s inverted conical shape enables favorable packing with conical lipids such as truncated OxPCs or lysolipids, thereby stabilizing planar bilayer organization [18]. Consequently, higher Chol content increases the likelihood of Chol–OxPC pairing, effectively segregating these conical species from the bulk phospholipid matrix and reducing their disruptive impact. While Chol is essential for maintaining membrane integrity and function, its concentration must remain tightly regulated within a narrow physiological range specific to each cell type.

We observed a stronger rigidifying effect for POVPC than PGPC in monounsaturated heterogeneous matrices, whereas no substantial changes in lipid packing were detected in polyunsaturated systems. Although this appears inconsistent with our previous observations in single-component POPC and PDPC bilayers, where PGPC exerted a stronger fluidizing effect than POVPC [19], these findings instead highlight the decisive role of lipid–lipid interactions in remodeling complex membrane architectures. The effects of oxidized phospholipids on membrane order are governed by the collective properties of the lipid matrix, including acyl chain unsaturation, Chol content, and phase organization. Within this framework, OxPCs do not act as intrinsically disordering agents; rather, their impact emerges from their interaction with the surrounding lipids. This principle is exemplified by lyso-PC, a conical lipid that reduces bending rigidity and promotes membrane flexibility in POPC bilayers by inducing positive curvature and disrupting lipid packing [20]. As the concentration of lyso-PC increases, the membrane becomes progressively less resistant to bending, demonstrating that lipid composition directly governs membrane mechanical stability and curvature behavior. Specifically, even small molar fractions of lyso-PC lead to a pronounced reduction in bending rigidity in pure POPC bilayers. In contrast, the opposite effect is observed in PDPC-enriched POPC membranes, where lyso-PC increases membrane rigidity due to the interplay between PDPC-induced membrane thinning and the tendency of lyso-PC to destabilize the lamellar phase [20,21,22]. Furthermore, membrane bending rigidity measurements provided complementary mechanical evidence that polyunsaturated lipid environments could fundamentally alter the way truncated OxPCs modulate membrane structure and mechanics. In more complex ternary raft-like mixtures, OxPCs might promote tighter packing of surrounding saturated lipids and Chol to reduce local free volume [18,23]. Even if OxPCs themselves are “defects”, they may preferentially partition near domain boundaries or in certain regions [24,25], thus altering domain dynamics (coalescence, line tension) in a way that increases order across the system [26,27]. Also, the presence of SM and Chol means that lipid packing constraints and cooperative interactions (tail–tail van der Waals, headgroup interactions, Chol ordering) dominate over the disordering effect of the truncated oxidized lipids [18]. Thus, the same OxPC molecules can disrupt a simple bilayer in L_d_ phase but trigger reorganization toward greater order in a raft-like bilayer in L_o_/L_d_ phase coexistence [28]. In addition, in single-component POPC or PDPC bilayers, the intrinsic molecular geometry of each OxPC dominates: the highly hydrated carboxyacyl PGPC adopts a strongly conical shape that disrupts interfacial packing and enhances bilayer disorder more effectively than POVPC, consistent with other studies about the effect of oxidized lipids on POPC lipid bilayers [29]. In contrast, in heterogeneous raft-like mixtures the membrane environment overrides the intrinsic disordering tendencies of the oxidized lipids. POVPC, with its less hydrated aldehyde group and truncated *sn-2* chain, interacts more favorably with Chol and partitions preferentially toward domain boundaries, where it can increase line tension and promote condensation of the SM/Chol-rich regions as reflected in model systems where oxidized PC analog stabilizes phase coexistence [30]. As a result, POVPC contributes to tighter packing and enhanced L_o_ phase stability in POPC-containing ternary mixtures. PGPC, however, is excluded from highly ordered SM/Chol regions due to its highly hydrated headgroup and therefore remains confined to the L_d_ phase, where its fluidizing effect becomes locally diluted and does not propagate into overall modifications of membrane order. This has been observed in model phase-separated membranes, where truncated oxidized lipids accumulate in disordered regions rather than in raft-like domains, and oxidized PC analogs show L_d_ preference in coexistence systems [31]. In polyunsaturated ternary mixtures, the extremely disordered and Chol-incompatible nature of the DHA-rich matrix further suppresses any ability of OxPCs to modulate packing effectively “absorbing” their structural perturbations [32]. Thus, the divergent effects of POVPC and PGPC on different membrane systems highlight that the OxPC-induced rigidifying or fluidizing effect is an emergent property arising from the interplay between oxidized lipid structure, Chol miscibility, domain organization, and the inherent mechanical compliance of the supporting lipid framework.

OxPCs stimulated the formation of raft domains with larger sizes, which supports the results obtained by other authors [33,34]. It has been reported that oxidative processes promote the formation of lipid raft domains in POPC:DPPC:Chol membranes [35]. They interpreted this effect as a result of the hydrophobic mismatch caused by the differences between the packing of oxidized and non-oxidized phospholipids. It has been found that, in an aqueous environment, hydrophobic tails cluster together to minimize the unfavorable exposure to water, so lipids must segregate according to their acyl chain length, which introduces lateral heterogeneity [4]. This thickness mismatch between longer and shorter fatty acids of the lipids governs domain size, with larger mismatches promoting the formation of larger domains [36], consistent with our observation of increased nanodomain size in polyunsaturated membranes. These larger but more dynamic raft-like domains in PDPC-containing membranes may provide extended yet flexible platforms for signaling complexes. Such domains may facilitate the transient recruitment of signaling proteins while preventing excessive stabilization and prolonged activation [37].

Membrane lateral organization plays a central role in regulating secretory phospholipase A_2_ (sPLA_2_) activity, which is governed primarily by the physicochemical properties of the lipid–water interface rather than by lipid composition and substrate concentration alone [38,39]. Classical structural and mechanistic studies established that sPLA_2_ catalysis occurs at the membrane interface and requires precise positioning of the enzyme relative to the lipid surface [40]. More recent work has refined this view by demonstrating that sPLA_2_ does not bind membranes in a single static configuration, but rather dynamically samples multiple orientations that are each functionally relevant for hydrolysis [41]. The present study directly complements these mechanistic insights by showing how membrane lipid composition, specifically FA unsaturation, SM, Chol and OxPCs, governs sPLA_2_ activity by modulating the physical landscape of the bilayer interface.

Cholesterol- and sphingolipid-rich L_o_ domains exhibit tight lipid packing and reduced interfacial hydration, which restrict enzyme penetration and suppress sPLA_2_ activity. In contrast, L_d_ regions provide enhanced lipid mobility and structural defects that favor enzyme binding and catalysis. The highest sPLA_2_ activity is observed at L_o_/L_d_ domain boundaries, where hydrophobic mismatch and lateral stress promote exposure of the *sn-2* acyl chain [39,42,43]. Dynamic membrane reorganization during inflammation or apoptosis therefore enhances sPLA_2_ activity by increasing membrane disorder [44].

In line with this work, we observed the lowest sPLA_2_ activity in membranes with the highest SM and Chol content, underscoring the inhibitory role of lipid order on enzyme function. From a mechanistic standpoint, such ordered membranes likely limit the ability of sPLA_2_ to adopt catalytically productive orientations on the bilayer surface. It was demonstrated that sPLA_2_ “wobbles” between three distinct membrane-bound positions, each associated with different steps of the hydrolysis process, including substrate binding, catalysis and product release. In highly ordered SM/Chol-rich membranes, this wobbling behavior is expected to be sterically and energetically constrained, thereby reducing overall enzymatic efficiency [41]. In monounsaturated POPC-containing membranes, no statistically significant differences in sPLA_2_ activity were observed between ternary mixtures with a POPC:SM ratio of 1:1 and Chol contents ranging from 50 to 100 mol%. This suggests that once a critical level of SM-induced order is reached, additional Chol does not further restrict enzyme activity in monounsaturated systems. The limited conformational flexibility of monounsaturated acyl chains creates a membrane environment in which sPLA_2_ binding modes are already constrained. This observation is consistent with the notion that membrane–enzyme interactions, rather than substrate chemistry alone, define the effective reaction rate [45]. In contrast, polyunsaturated PDPC-containing membranes exhibited a pronounced sensitivity to SM and Chol concentration. The lowest sPLA_2_ activity was observed in PDPC:SM:Chol mixture with an equimolar lipid ratio, corresponding to the most ordered membrane environment. Polyunsaturated fatty acids typically enhance lipid disorder and promote sPLA_2_ activity by increasing interfacial defects and facilitating enzyme penetration. However, the present results demonstrate that this intrinsic susceptibility can be suppressed when strong SM–Chol interactions dominate membrane organization. This finding aligns with the emerging concept that sPLA_2_ activity can be redirected or inhibited by membrane physical state, even in the presence of chemically favorable substrates [45].

The incorporation of oxidized phosphatidylcholines (POVPC and PGPC) appears to modulate sPLA_2_ activity through a combination of structural and chemical effects, reflecting the interplay between membrane organization and enzyme accessibility. In monounsaturated membranes, the inhibitory effect of POVPC can be rationalized by its ability to increase lipid order and stabilize L_o_ nanodomains, thereby reducing the density of interfacial packing defects required for efficient enzyme binding. In addition, the aldehyde functionality of POVPC may enhance headgroup cohesion, further limiting interfacial accessibility and amplifying its inhibitory effect [46]. In contrast, PGPC exerts a weaker ordering effect due to its highly hydrated carboxylated *sn-2* chain, which preserves interfacial defects and membrane hydration. Under conditions where L_o_/L_d_ phase boundaries are maximized, such as in equimolar mixtures, this structural context is expected to favor sPLA_2_ activity by increasing the availability of catalytically accessible sites. At these interfaces, PGPC-induced defects may facilitate enzyme binding and access to *sn-2* acyl chains. However, when domain boundaries are reduced or membrane order increases, this effect is diminished, indicating that PGPC-mediated modulation of sPLA_2_ activity is highly dependent on membrane organization. Furthermore, the presence of DHA-containing lipids contributes to maintaining interfacial disorder, which can attenuate the structural impact of oxidized phospholipids and preserve enzymatic activity [47]. Beyond structural effects, chemical factors may also play a role. The phosphocholine headgroups of POVPC and PGPC resemble that of sphingomyelin, a known interfacial inhibitor, suggesting that oxidized phospholipids may compete for enzyme binding sites and act as pseudo-inhibitors, thereby limiting access of sPLA_2_ to hydrolyzable phosphatidylcholine substrates [48,49]. Taken together, these considerations indicate that the effects of oxidized phospholipids on sPLA_2_ activity are not solely determined by their chemical structure, but emerge from their ability to reorganize the membrane interface and modulate the balance between lipid order, domain organization, and interfacial accessibility.

Oxidized phospholipids, including POVPC and PGPC, modulate sPLA_2_ activity, with important consequences for inflammation, tumor progression, and metabolic regulation. POVPC is generally associated with inhibitory effects on sPLA_2_, thereby limiting the production of inflammatory mediators and other bioactive lipids. In contrast, PGPC may sustain or enhance enzymatic activity, promoting pro-inflammatory or pro-tumorigenic signaling pathways [50,51,52]. Our findings demonstrate that the divergent effects of oxidized phospholipids are governed not only by their intrinsic chemical structure, but critically by membrane composition and lipid domain organization under physiological conditions. Consequently, OxPCs may act as key modulators of membrane-dependent signaling processes, particularly under conditions of oxidative stress [53,54].

## 4. Materials and Methods

### 4.1. Materials

**Figure 11:**
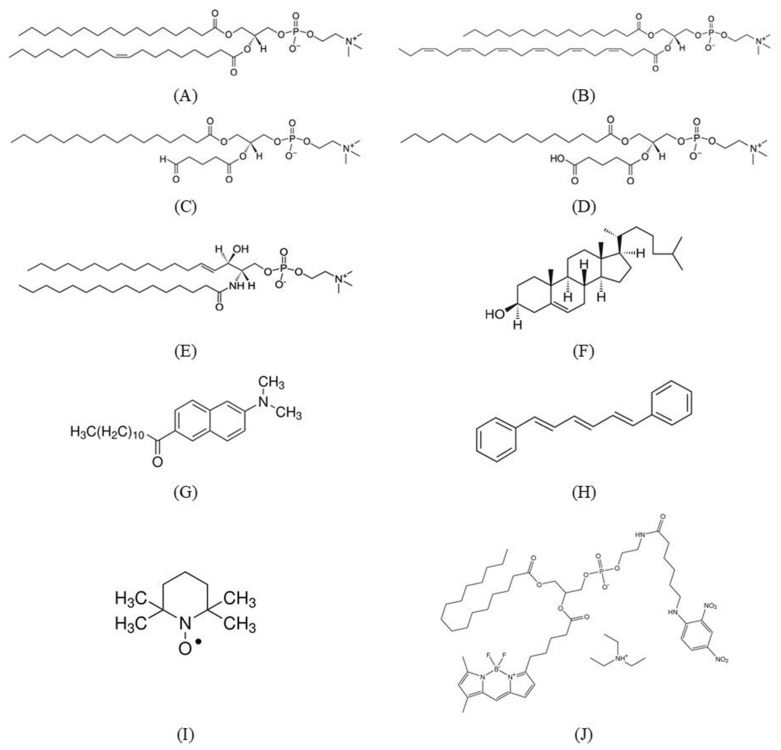
Chemical structures of the lipid species, fluorescent probes, and the enzyme substrate used in the study: (**A**) 1-palmitoyl-2-oleoyl-*sn*-glycero-3-phosphocholine (POPC); (**B**) 1-palmitoyl-2-docosahexaenoyl-*sn*-glycero-3-phosphocholine (PDPC); (**C**) 1-palmitoyl-2-(5′-oxo-valeroyl)-*sn*-glycero-3-phosphocholine (POVPC); (**D**) 1-palmitoyl-2-glutaryl-*sn*-glycero-3-phosphocholine (PGPC); (**E**) egg-yolk sphingomyelin (SM); (**F**) cholesterol (Chol) (Avanti Research, Alabaster, AL, USA); (**G**) 6-dodecanoyl-N, N-dimethyl-2-naphthylamine (Laurdan); (**H**) 1,6-Diphenyl-1,3,5-hexatriene (DPH); (**I**) 2,2,6,6-Tetramethylpiperidine 1-oxyl (TEMPO); and (**J**) N-((6-(2,4-dinitrophenyl) amino) hexanoyl)-2-(4,4-difluoro-5,7-dimethyl-4-bora-3a, 4a-diaza-s-indacene-3-pentanoyl)-1-hexadecanoyl-*sn*-glycero-3-phosphoethanolamine, triethylammonium salt (PED6).

The synthetic lipids used in our experiments were 1-palmitoyl-2-oleoyl-*sn*-glycero-3-phosphocholine (PC 16:0–18:1, POPC), 1-palmitoyl-2-docosahexaenoyl-*sn*-glycero-3-phosphocholine (PC 16:0–22:6, PDPC), 1-palmitoyl-2-(5′-oxo-valeroyl)-*sn*-glycero-3-phosphocholine (POVPC), 1-palmitoyl-2-glutaryl-*sn*-glycero-3-phosphocholine (PGPC), egg-yolk sphingomyelin (SM) and cholesterol (Chol). All these lipids were purchased from Avanti Research (Alabaster, AL, USA). The fluorescent probes 6-dodecanoyl-N, N-dimethyl-2-naphthylamine (Laurdan), 1,6-Diphenyl-1,3,5-hexatriene (DPH), the radical marker 2,2,6,6-Tetramethylpiperidine 1-oxyl (TEMPO) and the phospholipase A_2_ from European honey bee (*Apis mellifera*) venom (bvPLA_2_) were ordered from Sigma-Aldrich (St. Louis, MO, USA). The fluorogenic phospholipase A_2_ substrate N-((6-(2,4-dinitrophenyl) amino) hexanoyl)-2-(4,4-difluoro-5,7-dimethyl-4-bora-3a, 4a-diaza-s-indacene-3-pentanoyl)-1-hexadecanoyl-*sn*-glycero-3-phosphoethanolamine, triethylammonium salt (PED6) was obtained from ThermoFisher Scientific (Life Technologies Corporation, Waltham, MA, USA). The chemical structures of all synthetic substances investigated in the present study are shown in Figure 11.

### 4.2. Methods

#### 4.2.1. Liposome Preparation

Large unilamellar vesicles (LUVs) were formed by using the extrusion method. The lipid mixtures (1 mM lipid for Laurdan- and DPH-labeled LUVs and 0.1 mM lipid for the enzyme assay) were made from stock solutions in chloroform/methanol 1:1 (*v*/*v*). For fluorescence spectroscopy measurements of Laurdan and DPH-TEMPO quenching, the fluorescent probes were added during the preparation of each lipid mixture at 200:1 and 1000:1 lipid:probe molar ratios, respectively. The substrate PED6 (1 mM solution in absolute ethanol) for the fluorogenic PLA_2_ assay was also mixed with the initial lipid solutions at a PC:PED6 ratio of 10:1 (mol/mol). The organic solvents were then evaporated under a stream of oxygen-free dry nitrogen. For complete removal of the solvent, the lipid films were left under vacuum overnight. Heated at 60 °C, Tris buffer (10 mM Tris-HCl, 150 mM NaCl, 0.1 mM CaCl_2_·2H_2_O, pH 7.5) was then added to the dried lipid films. The suspensions were heated at 60 °C (5 min), then vortexed (1 min), sonicated in an ultrasonic bath Fisherbrand^®^ (1 min) and ice-cooled (5 min). These operations were repeated three times in order to provide the lipid mixing. The multilamellar vesicles (MLVs) obtained at this stage were then extruded with a LiposoFast small-volume extruder equipped with polycarbonate filters (Avestin, Ottawa, ON, Canada) as follows: 11 extrusions through 800 nm, followed by 21 extrusions through 100 nm filters. The samples were measured the next day.

#### 4.2.2. Fluorescence Spectroscopy of Laurdan-Labeled LUVs

The lipid packing of LUVs was evaluated by using the fluorescent marker Laurdan. It is incorporated into the membrane with the fluorescent naphthalene moiety situated at the level of the interface region (glycerol backbone), and the lauric acid tail anchored in the hydrophobic core of the bilayer. The naphthalene moiety possesses a dipole moment due to a partial charge separation between the 2-dimethylamino and the 6-carbonyl residues. This dipole moment increases upon excitation, causing the rearrangement of the surrounding water molecules [55]. In tightly packed membranes in liquid-ordered (L_o_) phase with reduced molecular mobility, the dipolar relaxation of water molecules is too slow to change the fluorescent emission, thus the emission spectra have only one peak at 440 nm in the blue range. However, in loosely packed membranes in liquid-disordered (L_d_) phase, where the lipid molecules have high rotational and lateral mobility, dipolar relaxation of water molecules occurs in the surroundings of the probe, which is reflected in a red-shift of about 50 nm in the steady-state emission spectrum of Laurdan, increasing the fluorescence at 490 nm [56]. Thus, Laurdan shows specific emission peaks at 440 and 490 nm that originate from lipid membranes in L_o_ and L_d_ phase, respectively [57]. The shift in the emission maximum can be quantified by the Generalized Polarization (GP) parameter as follows:

GP = (I_440_ − I_490_)/(I_440_ + I_490_),

where I_440_ and I_490_ are the emission intensities at 440 and 490 nm. Laurdan GP provides information on the hydration degree/lipid order in the polar head area near the glycerol backbone. Theoretically the values for the GP function go from −1.0 (least ordered) to +1.0 (most ordered); however, experimentally they range from −0.3 to 0.6 [55] both for pure lipids and for mixtures.

##### Laurdan Spectra Measurements

Steady-state fluorescence measurements were carried out with a FP-8300 spectrofluorometer (Jasco Inc., Easton, MD, USA) equipped with a Xenon arc lamp. Quartz cuvette was used. The vesicle suspensions were diluted to an overall lipid concentration of 0.5 mM. Heating temperature scans were performed from 20 °C to 60 °C, allowing sample equilibration for 5 min when the desired temperature was reached. The temperature in the cuvette holder was maintained using a water-circulating bath (Julabo, Seelbach, Germany). Excitation wavelength for Laurdan was 355 nm. The emission spectra were recorded from 390 to 600 nm in steps of 0.5 nm (bandwidths of 5 nm were chosen for both the excitation and emission monochromators) in triplicate and the calculated GP values were averaged. Analysis of the steady-state spectra was performed by using OriginPro 9.0.

To evaluate the OxPC effect on the lipid order, we calculated the relative GP change ΔGP/GP = ((GP_OxPC_ − GP)/GP) ∗ 100, where GP_OxPC_ denotes the value in the OxPC-containing sample, and GP stands for the same sample without OxPC (control) at physiological temperature of 37 °C (absolute GP values in Figure 3, Inset), ∆GP/GP (%), shown in Figure 4. Positive values correspond to rigidification and negative ones indicate fluidization of the lipid bilayers.

#### 4.2.3. DPH-TEMPO Fluorescence Spectroscopy

DPH-TEMPO fluorescence spectroscopy is developed for characterizing domains in the nanometer range using the probe TEMPO to quench the fluorescent marker DPH. By this method, the degree of lipid ordering can be determined, and the raft size can also be calculated. The heterocyclic compound TEMPO is an extremely stable nitroxyl radical widely used in biochemistry. DPH is one of the most used fluorescent molecules to study hydrophobic regions in membranes. The process in which the energy from an excited fluorophore molecule is transferred to an acceptor molecule—a “quencher” of the FL—without emitting a photon is called fluorescence resonance energy transfer (FRET). Initially, the donor fluorophore (DPH) absorbs energy upon excitation by light of a certain wavelength and transfers the excitation energy to the nearby chromophore (TEMPO)—the acceptor. Energy transfer is manifested in a decrease or quenching of the FL of the donor molecule, as well as a decrease in the lifetime of the excited state of the molecule [58]. DPH molecules are equally distributed in the two coexisting phases (L_o_ and L_d_) [59], while TEMPO, as a water-soluble molecule, diffuses into the L_d_ phase [60]. Therefore, at a less ordered membrane, TEMPO quenches DPH fluorescence (λ_exc_/λ_em_ (MetOH) = 350/452 nm), while at a more ordered state, the quenching is difficult [61]. The remaining DPH fluorescence in the presence of TEMPO (Q) was calculated according to

Q = F/F_0_,

where F and F_0_ are the FL intensities of DPH in the presence and absence of TEMPO, respectively. Q gives information about the degree of lipid ordering in the bilayer. High values of Q define weak quenching of the DPH fluorescence, indicating a highly ordered membrane. Conversely, low values result in a more disordered membrane.

##### Measurements of Fluorescence Quenching of DPH by TEMPO

Measurements were performed on a Synergy^TM^2 microplate reader (BioTek Instruments, Winooski, VT, USA). First, 0.2 mL of each sample (diluted to a final lipid concentration of 0.5 mM) was applied to the wells of the assay plate (Greiner, CELLSTAR^®^, Merck KGaA, Darmstadt, Germany). The experimental protocol included shaking the plate for 20 s, incubating for 10 min at 37 °C and recording the DPH fluorescence (F_0_) at excitation of 360 (±40) nm and emission of 460 (±40) nm. TEMPO (dissolved in ethanol) was then added to the samples at a final concentration of 2 mM. After incubation for 10 min at 37 °C, the DPH fluorescence remaining after quenching (F) was recorded in triplicate and the calculated Q values were averaged.

##### Calculation of the Domain Radius

Based on the calculated Q values for each lipid mixture, the radius of the L_o_ nanodomains in the membranes, R_dom_ (Å), can be evaluated according to [61]

R_dom_ = R_o_ ((Q − Q_PC_)/(Q_SM:Chol_ − Q_PC_)),

where R_o_ ≈ 4R_c_ ≈ 48 Å, at which about 50% quenching of DPH molecules is observed [62]. The effective quenching radius (R_c_) of the DPH molecule by the nitroxide group of the TEMPO is about 12 Å [14]. Q_PC_ and Q_SM:Chol_ are the values for residual DPH fluorescences of the “pure” lipid phases: PC as a model of the L_d_ phase, and SM:Chol as a model of the L_o_ phase [63]. The Q values stand for F/F_0_ ratios of the studied lipid systems, which were control ternary PC:SM:Chol and OxPC-containing quaternary PC:OxPC:SM:Chol mixtures, representing L_o_/L_d_ phase coexistence.

The relative R_dom_ change (%), ΔR/R = ((R_OxPC_ − R)/R) ∗ 100, provides information about the effect of OxPCs on L_o_ nanodomain size in PC:SM:Chol mixtures, where R_OxPC_ is defined as R_dom_ in the OxPC-containing sample, and R stands for R_dom_ in the same sample without OxPC (Figure 6(A2,B2)).

#### 4.2.4. Fluorogenic PLA_2_ Assay

PED6 is a phosphatidylethanolamine molecule with a fluorescent BODIPY dye-labeled *sn-2* acyl chain and a dinitrophenyl-label led polar group as a fluorescence quencher. Cleavage of the fluorescent fatty acid by sPLA_2_ eliminates the intramolecular action of the dinitrophenyl group, leading to an increase in fluorescence of BODIPY [64]. Accordingly, the increase in the FL signal corresponds to increasing sPLA_2_ activity.

##### Kinetic Measurements to Assess PLA_2_ Activity

Measurements were performed with a Synergy^TM^2 microplate reader (BioTek Instruments, Winooski, VT, USA) in a 96-well assay plate (Greiner, CELLSTAR^®^, Merck KGaA, Darmstadt, Germany) at 1 min time intervals (sensitivity = 65). Then, 192 µL of LUV samples (50 µM PC concentration) were aliquoted into wells and the plate was incubated at 37 °C for 10 min, shaken for 20 s, and FL emission intensities were read at 485 (±20) nm excitation and 528 (±20) nm emissions for 5 min, representing the baseline before bvPLA_2_ addition as a no-enzyme control. Immediately before addition to the samples, the enzyme was activated by transferring it to an assay buffer with a higher calcium content (10 mM Tris-HCl, 150 mM NaCl, 1 mM CaCl_2_, pH7.5). Then, 8 µL of PLA_2_ at a concentration of 2.5 µg/mL was added to the samples to obtain a final volume of 200 µL per well. The substrate (PC + PED6)/enzyme ratio was about 8000:1. The measurement protocol involves enzyme kinetic readings for 150 min, starting as soon as possible after the enzyme addition. FL changes were monitored in real time, as the hydrolysis of the fluorogenic substrate PED6 released BODIPY-labeled fatty acids. FL emission, directly proportional to enzyme activity, increased over time along kinetic curves with different slopes, which were used to calculate reaction rates. Initially, the reaction rate is approximately linear, then slows as the substrate is depleted, eventually reaching a steady-state plateau. Therefore, only the linear portion of the curve is used to determine enzymatic activity, producing a typical rectangular hyperbola-shaped kinetic curve [65]. The experiments were repeated 3 times, each of them including 4 measurements (12 kinetic curves for each of the studied lipid mixtures). Due to differences in relative fluorescent units (RFU) for each sample between individual experiments, the kinetic curve representing the hydrolysis of PED6 incorporated in the single-component control POPC samples was normalized to 1.0 at 150 min [19]. Kinetic curves of PED6 hydrolysis in control ternary PC:SM:Chol and OxPC-containing quaternary LUVs by bvPLA_2_ were recalculated according to the POPC sample.

## 5. Conclusions

The present results demonstrate that oxidized phospholipids induce distinct, composition-dependent structural and functional effects in lipid membranes. In monounsaturated POPC systems, OxPCs promote significant increases in membrane order and liquid-ordered (L_o_) domain size, reflecting substantial reorganization of lipid packing. In contrast, polyunsaturated PDPC membranes exhibit minimal changes in both membrane order and domain size, indicating a strong buffering capacity against oxidative perturbations and reduced membrane susceptibility to structural disruption.

Importantly, these structural differences are reflected in sPLA_2_ activity; however, this relationship is not direct or linear, revealing a clear decoupling between membrane physical properties and enzymatic response. In POPC membranes, OxPCs strongly modulate enzyme function, with POVPC consistently acting as a potent inhibitor under all conditions, whereas PGPC displays a Chol-dependent dual behavior, switching from inhibition at low Chol to pronounced activation at high Chol. This PGPC-induced activation is markedly attenuated in PDPC membranes, highlighting a selective protective effect of DHA-rich environments. In contrast, the inhibitory effect of POVPC remains largely unaffected by lipid unsaturation.

These findings provide important insight into the biological consequences of lipid oxidation. While oxidative stress is generally associated with pro-inflammatory responses, our results demonstrate that the effects of oxidized lipids are not uniform but depend on both lipid structure and membrane context. Specifically, POVPC may suppress sPLA_2_-mediated inflammatory signaling, whereas PGPC can promote enzyme activation under conditions of high membrane order. The selective attenuation of PGPC-induced activation in DHA-containing membranes suggests that polyunsaturated lipids may play a protective role by limiting inflammation driven by specific oxidized lipid species.

Together, these results establish a mechanistic link between lipid oxidation, membrane organization, and the regulation of pro-inflammatory enzyme activity. They further demonstrate that membrane susceptibility to oxidative damage is governed by the collective properties of the lipid matrix rather than by the presence of oxidized lipids alone, emphasizing that membrane composition is a key determinant of cellular responses to oxidative stress. More broadly, this work identifies membrane organization as an active regulatory element in oxidative stress responses, rather than a passive structural background, with important implications for understanding and targeting membrane-driven processes in disease.

## Figures and Tables

**Table 1:** Effect of oxidized phospholipids (OxPCs) at the highest studied concentration and lipid unsaturation on sPLA_2_ activity (ΔF/F, %), membrane order (ΔGP/GP, %), and L_o_ domain size (ΔR/R, %) under low- and high-cholesterol conditions in ternary PC:SM:Chol mixtures.

	sPLA_2_		Lipid Order		L_o_ Domain Size	
OxPC	POPC	PDPC	POPC	PDPC	POPC	PDPC
POVPC (low Chol)	−85	−65	+48	0	+48	+10
PGPC (low Chol)	−75	−40	+20	0	+28	−5
POVPC (high Chol)	−80	−60	+18	0	+35	+7
PGPC (high Chol)	+350	+60	+20	0	+32	+9

## Data Availability

The original contributions presented in this study are included in the article/Supplementary Material. Further inquiries can be directed to the corresponding author.

## Supplementary Materials

The following supporting information can be downloaded at: https://www.mdpi.com/article/10.3390/molecules31081298/s1, Figure S1: Kinetic curves of hydrolysis of PED6 incorporated in control ternary monounsaturated POPC:SM:Chol (green font) LUVs (A) and polyunsaturated PDPC:SM:Chol (red font) ones (B) by sPLA_2_ at 37 °C. PC/PED6 and (PC + PED6)/enzyme ratios were 10:1 mol/mol and 8000:1 mol/mol. (1) FL = (F_530_/F_530, initial_) − 1, where F_530_ was the fluorescence intensity at 530 nm at time t, whereas F_530, initial_ represented the sample fluorescence intensity before bvPLA_2_ addition (at t = 0 min). FL signal increase was correspondent to sPLA_2_ activity elevation. FL values were presented of every 5 min for clarity. The data represent means ± SD from 3 experiments as each sample was measured 4 times (n = 12). (2) One-Way ANOVA analysis was performed to compare PC:SM:Chol samples at different molar ratios at each time point (5, 10, 15, 20, 30, 90 and 150 min) of the fluorogenic assay. Minor ticks on the X-axis were used to separate the comparison groups for clarity. The population data followed a normal Gaussian distribution. Groups sharing at least one common letter are not significantly different from each other, whereas groups labeled with different letters are significantly different. Figure S2: The reaction rate of hydrolysis of PED6 incorporated in control ternary monounsaturated POPC:SM:Chol (green font) and polyunsaturated PDPC:SM:Chol (red font) LUVs by sPLA_2_ at 37 °C. Linear regression of the initial part of the kinetic curves of normalized fluorescence intensity, FL, at 530 nm (FL = F_530_/F_530, initial_ − 1) = *a* + *b*t, where *a* and *b* denote the y-intercept (A) and the slope (B), respectively. The data represent means ± SD from 3 experiments as each sample was measured 4 times (n = 12). (A) The intercept yielded the FL value at t = 0 min. (B) The enzymatic reaction rate, dFL/dt (min^−1^), was determined as the slope of the kinetic curves. One-Way ANOVA analysis was performed. The population data followed a normal Gaussian distribution. Groups sharing at least one common letter are not significantly different from each other, whereas groups labeled with different letters are significantly different. Figure S3: Hydrolysis of PED6 incorporated in control monounsaturated (green font) and polyunsaturated (red font) ternary PC:SM:Chol LUVs (100:50:50, 100:100:50, 100:100:100) and OxPC-containing quaternary ones (OxPC = 30 mol% POVPC or PGPC) by sPLA_2_ at 37 °C at different times (5, 10, 15, 20, 30, 90 and 150 min) of the fluorogenic assay. PC/PED6 and (PC + PED6)/enzyme ratios were 10:1 mol/mol and 8000:1 mol/mol. FL = (F_530_/F_530, initial_) − 1, where F_530_ was the fluorescence intensity at 530 nm at time t, whereas F_530, initial_ represented the sample fluorescence intensity before bvPLA_2_ addition (at t = 0 min). FL signal increase was correspondent to sPLA_2_ activity elevation. One-Way ANOVA analysis was performed to compare control ternary samples with OxPC-containing quaternary ones, as well as POVPC-containing samples with PGPC-containing ones, at each time point (5, 10, 15, 20, 30, 90, and 150 min) of the fluorogenic assay. Minor ticks on the X-axis were used to separate the comparison groups for clarity. The population data followed a normal Gaussian distribution. Groups sharing at least one common letter are not significantly different from each other, whereas groups labeled with different letters are significantly different.

## Abbreviations

OxPLs	oxidized phospholipids
OxPCs	oxidized phosphatidylcholines
POVPC	1-palmitoyl-2-(5′-oxo-valeroyl)-*sn*-glycero-3-phosphocholine
PGPC	1-palmitoyl-2-glutaryl-*sn*-glycero-3-phosphocholine
sPLA_2_	secretory phospholipase A_2_
Lyso-PC	lysophosphatidylcholine
POPC	1-palmitoyl-2-oleoyl-*sn*-glycero-3-phosphocholine
PDPC	1-palmitoyl-2-docosahexaenoyl-*sn*-glycero-3-phosphocholine
L_o_	liquid-ordered
L_d_	liquid-disordered
GPLs	glycerophospholipids
SLs	sphingolipids
Chol	cholesterol
SM	sphingomyelin
PLs	phospholipids
PCs	phosphatidylcholines
FA	fatty acid
PUFA	polyunsaturated fatty acid
DHA	docosahexaenoic acid
PAPC	1-palmitoyl-2-arachidonoyl-phosphatidylcholine
OxLDLs	oxidized low density lipoproteins
OA	oleic acid
LUVs	large unilamellar vesicles
GP	generalized polarization
R_dom_	radius of the L_o_ nanodomains
FL	fluorescence
