# Supplementary material for: Oxidized Phosphatidylcholines Regulate Secretory Phospholipase A2 Through Membrane Nanodomain Remodeling
Source: Molecules. 2026 Apr 16;31(8):1298. doi: 10.3390/molecules31081298 (PMC13118827; doi:10.3390/molecules31081298)
Supplement: Supplementary file 1 [file molecules-31-01298-s001.zip › molecules-4212996-supplementary.pdf]

Supplementary Data

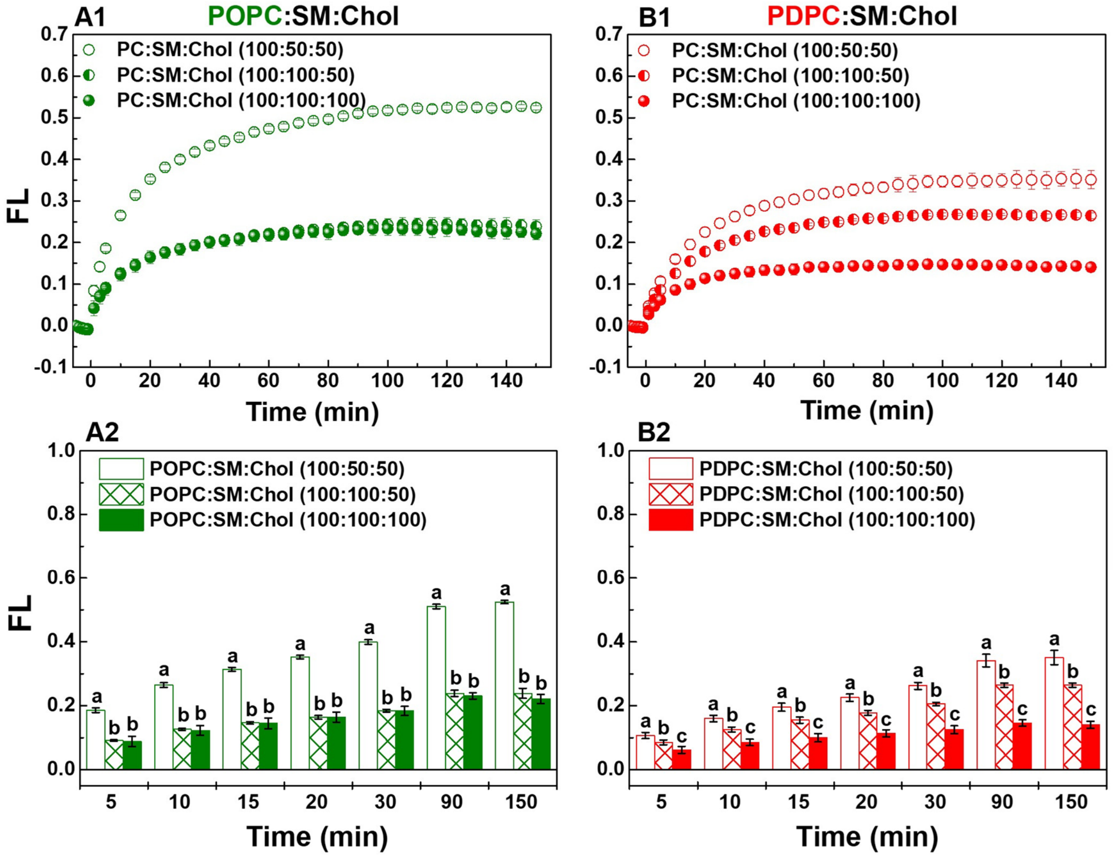

Figure S1. Kinetic curves of hydrolysis of PED6 incorporated in control ternary monounsaturated POPC:SM:Chol (green font) LUVs (A) and polyunsaturated PDPC:SM:Chol (red font) ones (B) by sPLA$_2$ at 37°C. PC/PED6 and (PC + PED6)/enzyme ratios were 10:1 mol/mol and 8000:1 mol/mol. (1) FL = (F_{530}/$F_{530,\ initial}$) – 1, where F_{530} was the fluorescence intensity at 530 nm at time t, whereas $F_{530,\ initial}$ represented the sample fluorescence intensity before bvPLA$_2$ addition (at t = 0 min). FL signal increase was correspondent to sPLA$_2$ activity elevation. FL values were presented of every 5 min for clarity. The data represent means ± SD from 3 experiments as each sample was measured 4 times (n=12). (2) One-Way ANOVA analysis was performed to compare PC:SM:Chol samples at different molar ratios at each time point (5, 10, 15, 20, 30, 90 and 150 minutes) of the fluorogenic assay. Minor ticks on the X-axis were used to separate the comparison groups for clarity. The population data followed a normal Gaussian distribution. Groups sharing at least one common letter are not significantly different from each other, whereas groups labeled with different letters are significantly different.

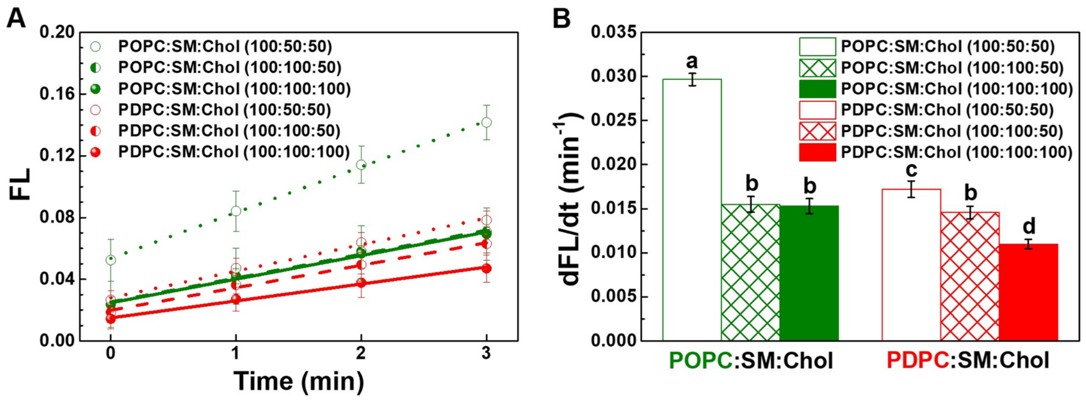

Figure S2. The reaction rate of hydrolysis of PED6 incorporated in control ternary monounsaturated POPC:SM:Chol (green font) and polyunsaturated PDPC:SM:Chol (red font) LUVs by $sPLA_2$ at 37°C. Linear regression of the initial part of the kinetic curves of normalized fluorescence intensity, FL, at 530 nm ($FL = F_{530}/F_{530,\ initial} - 1$) = $a + bt$, where a and b denote the y-intercept (A) and the slope (B), respectively. The data represent means ± SD from 3 experiments as each sample was measured 4 times (n=12). (A) The intercept yielded the FL value at t = 0 min. (B) The enzymatic reaction rate, dFL/dt (min^{-1}), was determined as the slope of the kinetic curves. One-Way ANOVA analysis was performed. The population data followed a normal Gaussian distribution. Groups sharing at least one common letter are not significantly different from each other, whereas groups labeled with different letters are significantly different.

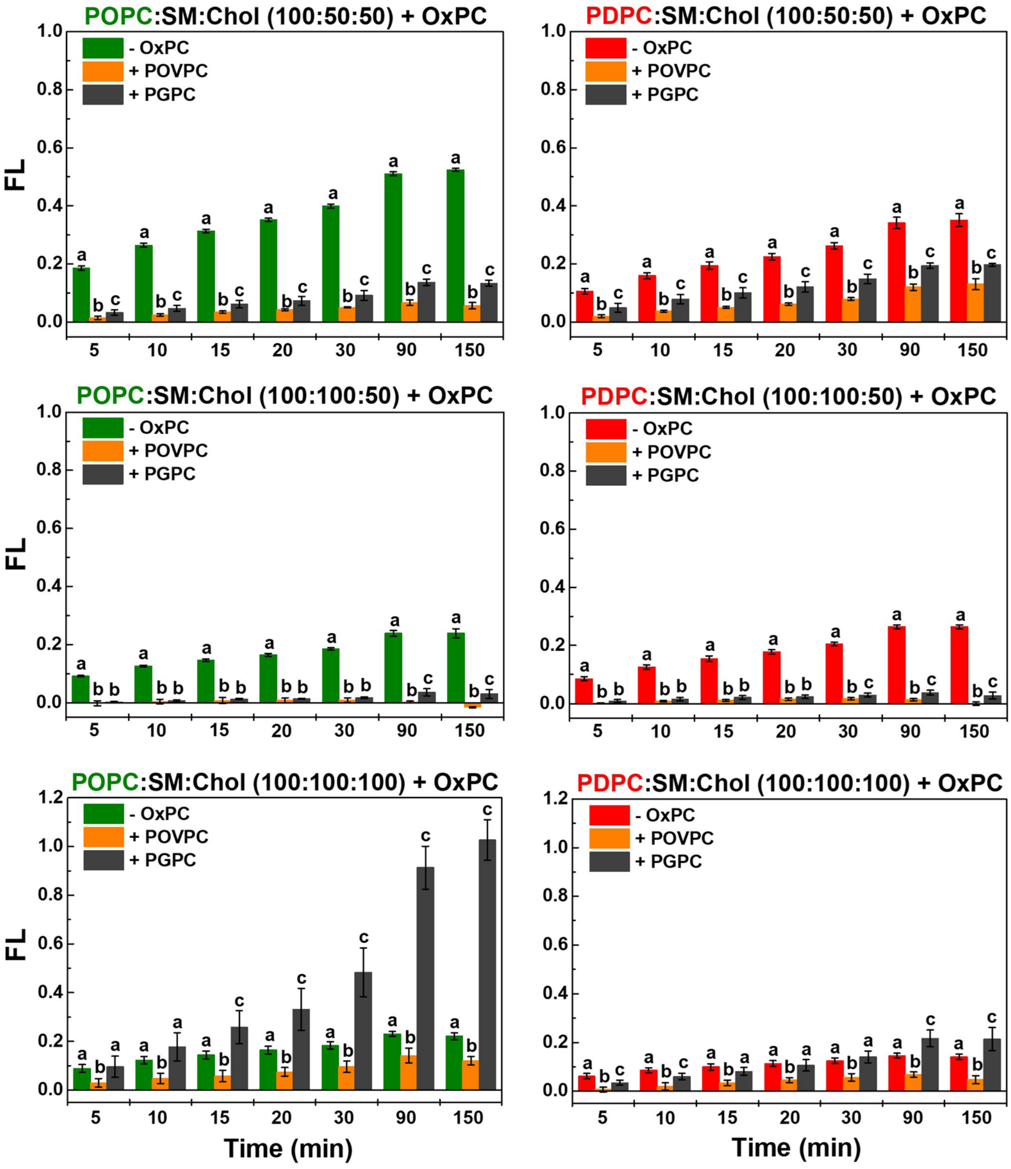

Figure S3. Hydrolysis of PED6 incorporated in control monounsaturated (green font) and polyunsaturated (red font) ternary PC:SM:Chol LUVs (100:50:50, 100:100:50, 100:100:100) and OxPC-containing quaternary ones (OxPC = 30 mol% POVPC or PGPC) by $sPLA_2$ at 37°C at different times (5, 10, 15, 20, 30, 90 and 150 min) of the fluorogenic assay. PC/PED6 and (PC + PED6)/enzyme ratios were 10:1 mol/mol and 8000:1 mol/mol. FL = ($F_{530}/F_{530,\ initial}$) − 1, where F_{530} was the fluorescence intensity at 530 nm at time t, whereas $F_{530,\ initial}$ represented the sample fluorescence intensity before $bvPLA_2$ addition (at t = 0 min). FL signal increase was correspondent to $sPLA_2$ activity elevation. One-Way ANOVA analysis was performed to compare control ternary samples with OxPC-containing quaternary ones, as well as POVPC-containing samples with PGPC-containing ones, at each time point (5, 10, 15, 20, 30, 90, and 150 minutes) of the fluorogenic assay. Minor ticks on the X-axis were used to separate the comparison groups for clarity. The population data followed a normal Gaussian distribution. Groups sharing at least one common letter are not significantly different from each other, whereas groups labeled with different letters are significantly different.
